# Gums as Macromolecular Crowding Agents in Human Skin Fibroblast Cultures

**DOI:** 10.3390/life14040435

**Published:** 2024-03-25

**Authors:** Salome Guillaumin, Mehmet Gurdal, Dimitrios I. Zeugolis

**Affiliations:** 1 Regenerative, Modular & Developmental Engineering Laboratory (REMODEL) and Science Foundation Ireland (SFI) Centre for Research in Medical Devices (CÚRAM), Biomedical Sciences Building, University of Galway, H91 TK33 Galway, Ireland; s.guillaumin1@universityofgalway.ie (S.G.); mehmet.gurdal88@gmail.com (M.G.); 2 Regenerative, Modular & Developmental Engineering Laboratory (REMODEL), Charles Institute of Dermatology, Conway Institute of Biomolecular & Biomedical Research and School of Mechanical & Materials Engineering, University College Dublin (UCD), D04 V1W8 Dublin, Ireland

**Keywords:** gums, macromolecular crowding, skin fibroblasts, extracellular matrix deposition

## Abstract

Even though tissue-engineered medicines are under intense academic, clinical, and commercial investigation, only a handful of products have been commercialised, primarily due to the costs associated with their prolonged manufacturing. While macromolecular crowding has been shown to enhance and accelerate extracellular matrix deposition in eukaryotic cell culture, possibly offering a solution in this procrastinating tissue-engineered medicine development, there is still no widely accepted macromolecular crowding agent. With these in mind, we herein assessed the potential of gum Arabic, gum gellan, gum karaya, and gum xanthan as macromolecular crowding agents in WS1 skin fibroblast cultures (no macromolecular crowding and carrageenan were used as a control). Dynamic light scattering analysis revealed that all macromolecules had negative charge and were polydispersed. None of the macromolecules affected basic cellular function. At day 7 (the longest time point assessed), gel electrophoresis analysis revealed that all macromolecules significantly increased collagen type I deposition in comparison to the non-macromolecular crowding group. Also at day 7, immunofluorescence analysis revealed that carrageenan; the 50 µg/mL, 75 µg/mL, and 100 µg/mL gum gellan; and the 500 µg/mL and 1000 µg/mL gum xanthan significantly increased both collagen type I and collagen type III deposition and only carrageenan significantly increased collagen type V deposition, all in comparison to the non-macromolecular crowding group at the respective time point. This preliminary study demonstrates the potential of gums as macromolecular crowding agents, but more detailed biological studies are needed to fully exploit their potential in the development of tissue-engineered medicines.

## 1. Introduction

Cell therapies are continuously gaining pace in reparative and regenerative medicine. To put things into perspective, the global cell therapy market size was valued at USD 7.8 billion in 2019 and is expected to reach USD 48.1 billion by 2027, registering a compound annual growth rate of 25.6% from 2020 to 2027 [1]. A ClinicalTrials.gov search (term searched: cell therapy in other terms; date of search: 7 August 2023) returned 59,567 studies at various stages (e.g., completed, recruiting, enrolling by invitations, not yet recruiting). A PubMed search (term searched: cell therapy in title only; date of search: 7 August 2023) returned 416 published documents when only clinical trial and randomised controlled trial article types were included, whilst 9268 published documents were returned when no article type restrictions were applied (other than cell therapy in the title only). Despite these massive investment and academic outputs in the field, only 29 cellular and gene therapy products have been licenced from the FDA’s Office of Tissues and Advanced Therapies [2]. The limiting factors with respect to direct cell injections are the poor cell survival and localisation at the implantation site [3,4], whilst tissue engineered medicines require prolonged in vitro culture periods for the development of implantable products, which are associated with cell phenotypic drift [5,6] and extremely high manufacturing costs [7,8,9,10,11], making the therapies prohibitively expensive for healthcare providers and inaccessible to patients.

Macromolecular crowding (MMC) via volume exclusion (i.e., two molecules cannot be at the same place at a given time point) significantly reduces molecular diffusion in highly crowded and structureless solutions, resulting in significantly increased kinetics of biochemical reactions and biological processes [12,13,14,15,16,17]. In a diverse range of permanently differentiated and mesenchymal stomal cell culture systems, MMC (i.e., the addition of macromolecules in culture media) has been shown to enhance and accelerate extracellular matrix (ECM) deposition [18,19]. As a direct consequence, tissue engineered medicines [20] and cell derived matrices for cell expansion [21], drug discovery [22], and reparative [23] purposes with superior to the state-of-the-art functionality can now be developed using only a fraction cells and time that traditional approaches require.

Despite the notable advances in MMC technology in cell culture context, there is no widely accepted MMC agent, and there are remarkable differences in the effectiveness of those that have been used to date. For example, although polysucrose [24,25,26,27], hyaluronic acid [28], dextran sulphate [29,30,31,32], polyvinylpyrrolidone [33], polysodium-4-styrene sulfonate [34], and cocktails thereof (e.g., dextran sulphate and polysucrose [35]) have shown potential as MMC agents, none has come close to the efficacy and efficiency of carrageenan (CR), largely attributed to its negative charge and polydispersity [36,37]. Considering though the rather questionable direct or indirect association of CR with colitis [38,39,40] that may restrict its use in biomedicine, despite its regulatory history, it is imperative to identify alternative MMC agents with a clear regulatory clearance pathway.

Gums are natural polysaccharides, made of different sugars, with an established history in the food sector as thickening, emulsifying, and stabilising agents [41,42,43]. In biomedicine, gum Arabic (GA) has been used as part of delivery vehicles loaded with anti-cancer [44] and anti-bacterial [45] therapeutics. Gum gellan (GG) has been used as a scaffold for skin [46], intervertebral disc [47], and dental [48] applications. Gum karaya (GK) has also been used as part of drug delivery vehicles [49,50]. Gum xanthan (GX) has been used extensively in the development of skin substitutes [51,52]. Despite this extensive use of gums in the biomedical field, their potential as MMC agents has yet to be assessed. Thus, herein, we ventured to assess the influence of these gums as MMC agents in WS1 skin fibroblast cultures. All cultures were conducted in the presence of L-ascorbic acid; essentially, ascorbic acid increases collagen synthesis/secretion [53,54,55,56], and the subsequent application of MMC increases collagen and associated ECM deposition.

## 2. Materials and Methods

### 2.1. Materials

CR, GA, GG, GK, and GX (Table S1 provides their properties) were purchased from Sigma-Aldrich (Arklow, Ireland). Tissue culture plasticware were purchased from Sarstedt (Dublin, Ireland) and NUNC (Roskilde, Denmark). All other chemicals, cell culture media, and reagents were purchased from Sigma-Aldrich (Arklow, Ireland), unless otherwise stated. WS1 skin fibroblasts (ATCC-CRL-150) were purchased from ATCC, London, UK. WS1 is a diploid fibroblast cell isolated from the skin of a black female donor (age: 12 weeks gestation) with a doubling potential of 67 population doublings. WS1 [36] and WI38 [29,31,36] (diploid fibroblast cell isolated from the lung of a white female donor (age: 3 months gestation) with doubling potential of 50 ± 10 population doublings) are used extensively to assess the potential of macromolecules as MMC agents, with data obtained being similar to those of permanently differentiated (e.g., human dermal [37], corneal [57], and tendon [58] fibroblasts) and mesenchymal stromal (e.g., human bone marrow [59], adipose-derived [60], and umbilical cord [61] mesenchymal stromal cells) cell populations.

### 2.2. Solubility Assessment

Solubility was assessed in standard cell culture media composed of Dulbecco’s modified Eagle’s medium high glucose (Gibco™, ThermoFisher Scientific, Dublin, Ireland), 10% foetal bovine serum, 1% penicillin and streptomycin (10,000 units penicillin and 10 mg streptomycin per mL), and 100 µM of L-ascorbic acid 2-phosphate sesquimagnesium salt hydrate. GG and GK were first heated at 90 °C. To identify working concentrations, first, GA, GG, GK, and GX were suspended in media at concentrations ranging from 25 µg/mL to 25,000 µg/mL. The solutions were left for 72 h on an orbital shaker at 37 °C at 150 rpm. Solutions with insoluble matter or visibly viscous were excluded from further analysis. After identification of the highest fully soluble concentration (Table S2), four concentrations were selected for each molecule (500, 1000, 2500, and 5000 μg/mL GA; 25, 50, 75, and 100 μg/mL GG; 25, 50, 75, and 100 μg/mL GK; and 50, 100, 500, and 1000 μg/mL GX) for subsequent analysis.

### 2.3. Dynamic Light Scattering Assessment

Hydrodynamic radius, polydispersity index, and zeta potential of CR and all gums at their respective concentrations were assessed using dynamic light scattering (Zetasizer ZS 90, Malvern Instrument, Malvern, UK). All solutions were prepared in ultrapure water. Fractional volume occupancy was calculated using the obtained values of a hydrodynamic radius for the respective molecules, as has been described before [62].

### 2.4. Cell Culture

Cells were cultured in Dulbecco’s modified Eagle’s medium high glucose (Gibco™, ThermoFisher Scientific, Dublin, Ireland) supplemented with 10% foetal bovine serum and 1% penicillin and streptomycin (10,000 units penicillin and 10 mg streptomycin per ml) at 37 °C in a humidified atmosphere of 5% CO_2_. Cells were used at passage 6 (were passaged in sub-confluent cultures, 70–80%). For MMC experiments, cells were seeded at 25,000 cells/cm^2^ density and were allowed to attach for 24 h. Subsequently, the media were changed with media containing 100 µM of L-ascorbic acid 2-phosphate sesquimagnesium salt hydrate and the various gums at the concentrations identified from the solubility assessment. Media with 100 µM of L-ascorbic acid 2-phosphate sesquimagnesium salt hydrate (-MMC) and media with 100 µM of L-ascorbic acid 2-phosphate sesquimagnesium salt hydrate and 75 μg/mL CR were used as the control. Media were changed every 2 days.

### 2.5. Cell Morphology Assessment

To evaluate the influence of different MMC conditions on cell morphology, an inverted microscope (Leica Microsystem, Wetzlar, Germany) was used. Phase contrast images were captured at different time points (3, 5, 7 days) and were processed using ImageJ software (https://imagej.net/ij/, NIH, Bethesda, MD, USA).

### 2.6. Cell Viability Assessment

At the various time points (3, 5, 7 days), calcein AM (ThermoFisher Scientific, Dublin, Ireland) and ethidium homodimer I (ThermoFisher Scientific, Dublin, Ireland) stainings were performed, as per the manufacturer’s protocol, to assess the influence of the different MMC agents on cell viability. Briefly, cells were washed with Hank’s Balanced Salt Solution, and a solution of calcein AM (4 μM in Hank’s Balanced Salt Solution) and ethidium homodimer I (2 μM in Hank’s Balanced Salt Solution) was added. Cells were incubated at 37 °C and 5% CO_2_ for 30 min, after which, fluorescence images were captured with an Olympus IX-81 inverted fluorescence microscope (Olympus Corporation, Tokyo, Japan) and processed using ImageJ software (NIH, Bethesda, MD, USA).

### 2.7. Cell Proliferation Assessment

At the various time points (3, 5, 7 days), the Quant-iT™ PicoGreen™ dsDNA Assay Kit (ThermoFisher Scientific, Dublin, Ireland) was used as per the manufacturer’s protocol to assess the influence of the different MMC agents on cell proliferation. Briefly, cells were washed with Hank’s Balanced Salt Solution, DNase-free water was added, and then the samples were frozen at −80 °C and subjected to three cycles of freeze–thawing to lyse the cells. Subsequently, PicoGreen^®^ working solution was added to the samples, and they were incubated at room temperature for 30 min, being protected from light. Fluorescence was measured at excitation and emission wavelengths of 480 nm and 520 nm, respectively, using a Varioskan Flash Multimode Reader (ThermoFisher Scientific, Dublin, Ireland). The obtained values were normalised to the standard curve, which was generated with a series of known DNA stock solutions at different concentrations (0, 5, 10, 25, 50, 100, 500, and 1000 ng/mL).

### 2.8. Cell Metabolic Activity Assessment

At the different time points (3, 5, 7 days), the alamarBlue^®^ assay (ThermoFisher Scientific, Dublin, Ireland) was used as per the manufacturer’s instructions to evaluate the influence of the various MMC agents on cell metabolic activity. Briefly, at each time point, cells were washed with Hank’s Balanced Salt Solution, and a 10% alamarBlue^®^ solution in Hank’s Balanced Salt Solution was added to the cells. Cells were incubated at 37 °C and 5% CO_2_ for 4 h, and absorbance was measured at excitation and emission wavelengths of 550 nm and 595 nm, respectively, with a Varioskan Flash Spectral scanning Multimode reader (ThermoFisher Scientific, Dublin, Ireland). Cell metabolic activity was expressed as % reduction of the alamarBlue^®^ dye and was normalised to the -MMC control group.

### 2.9. Collagen Deposition via Electrophoresis Assessment

Sodium dodecyl sulphate polyacrylamide gel electrophoresis (SDS-PAGE) was conducted to assess deposited collagen, as has been previously described [63]. Briefly, at the various time points (3, 5, 7 days), culture media were aspirated, and cell layers were briefly washed with Hank’s Balanced Salt Solution. Cell layers were then digested with pepsin from porcine gastric mucosa at 0.1 mg/mL in 0.05 M acetic acid (ThermoFisher Scientific, Dublin, Ireland) at 37 °C for 2 h under agitation. After digestion, cell layers were scraped and neutralised with 1 N sodium hydroxide. A total of 8 μL of cell layer solution or standard (100 μg/mL collagen type I, Symatese Biomateriaux, Chaponost, France) was mixed with 34 μL of deionised water and 18 μL of in-house 5x sample buffer, made of chemical purchased from Bio-Rad Laboratories, UK. The solution was vortexed and denatured for 5 min at 95 °C. A total of 10 μL of this solution was loaded per gel (3% stacking gel and 5% separation gel) lane and they were analysed (50 V for 30–40 min and 120 V 50–60 min) under non-reducing conditions with a Mini-Protean^®^ 3 electrophoresis system (Bio-Rad Laboratories, Watford, UK). Staining of the protein bands was performed with Pierce™ Silver Stain kit (ThermoFisher Scientific, Dublin, Ireland) following the manufacturer’s instructions. To quantify the deposited collagen type I, the relative densities of collagen α1(I) and α2(I) chains were evaluated with ImageJ and compared to the α1(I) and α2(I) chain band densities of the standard collagen type I.

### 2.10. Collagen Deposition via Immunofluorescence Assessment

At each time point (3, 5, 7 days), the cell layers were briefly washed with phosphate-buffered saline and fixed with 4% paraformaldehyde for 20 min at room temperature. Then, the cell layers were washed again, and non-specific site interactions were blocked with 5% bovine serum albumin in phosphate-buffered saline for 30 min. The cell layers were then incubated overnight at 4 °C with the primary antibodies (rabbit anti-collagen type I, NB600-408, Novus Biologicals, Centennial, CO, USA; rabbit anti-collagen type III, ab7778, Abcam, Cambridge, UK; rabbit anti-collagen type V, ab7046, Abcam, Cambridge, UK; all 1 to 200 dilution in phosphate-buffered saline), then were washed 3 times with phosphate-buffered saline and then incubated for 30 min at room temperature with the secondary antibody (AlexaFluor^®^ 488 donkey anti-rabbit, ThermoFisher Scientific, Dublin, Ireland; 1 to 400 dilution in phosphate-buffered saline). Nuclei were counterstained with Hoechst 33342 Fluorescent Stain (1 to 2000 dilution in phosphate-buffered saline, Invitrogen™, ThermoFisher Scientific, Dublin, Ireland). Fluorescent images were obtained using an inverted fluorescent microscope Olympus IX 81 (Olympus Corporation, Tokyo, Japan). Images (three images per group and three fields of view per image) were processed using the ImageJ software (NIH, Bethesda, MD, USA). Relative fluorescent intensity was normalised to the cell number.

### 2.11. Statistical Analysis

Data are expressed as mean ± standard deviation. Biological experiments were conducted in three independent experiments with one to four replicates per independent experiment. Statistical analysis was performed using SPSS (https://www.ibm.com/products/spss-statistics, IBM, Chicago, IL, USA) software for dynamic light scattering, cell proliferation, cell metabolic activity, and electrophoresis. For immunofluorescence, statistical analysis was conducted using Prism 8 (GraphPad, San Diego, CA, USA). One-way analysis of variance (ANOVA) was used for multiple comparisons, and the LSD post hoc test was used for pairwise comparisons when the group distributions were normal and variances of populations were equal. When either or both assumptions were violated, non-parametric analysis was conducted using the Kruskal–Wallis test for multiple comparisons and the Mann–Whitney test for pairwise comparisons. Results were considered statistically significant when *p* < 0.05.

## 3. Results

### 3.1. Zeta Potential, Polydispersity, Hydrodynamic Radius, and Fractional Volume Occupancy Assessment of the MMC Agents Used

**Figure 1:**
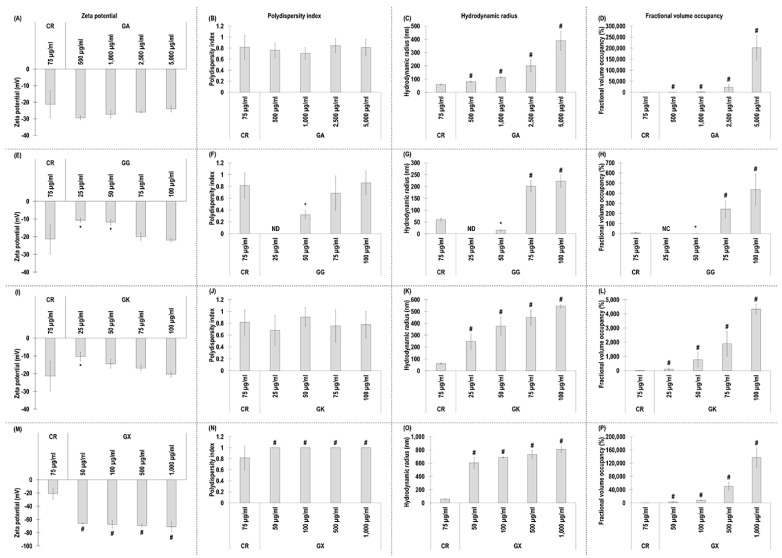
Zeta potential (mV), polydispersity index, hydrodynamic radius (nm), and fractional volume occupancy (%) analyses (via dynamic light scattering) of the MMC agents (carrageenan (CR) at 75 μg/mL; gum Arabic (GA) at 500, 1000, 2500, and 5000 μg/mL; gum gellan (GG) at 25, 50, 75, and 100 μg/mL; gum karaya (GK) at 25, 50, 75, and 100 μg/mL; and gum xanthan (GX) at 50, 100, 500, and 1000 μg/mL) assessed. # Indicates significantly (*p* < 0.05) higher population to CR. * Indicates significantly (*p* < 0.05) lower population to CR. ND stands for not detected. NC stands for not calculated. N = 3–6.

All GA concentrations exhibited similar (*p* > 0.05) to CR negative charge (Figure 1A) and polydispersity index (Figure 1B). All GA concentrations exhibited significantly (*p* < 0.05) higher in comparison to the CR hydrodynamic radius (Figure 1C) and % fraction volume occupancy (Figure 1D).

With respect to GG, the 25 µg/mL and 50 µg/mL concentrations exhibited significantly (*p* < 0.05) lower than CR, and the 75 µg/mL and 100 µg/mL concentrations exhibited similar (*p* > 0.05) to the CR negative charge (Figure 1E) and polydispersity index (Figure 1F). The 25 µg/mL and 50 µg/mL GG concentrations exhibited significantly (*p* < 0.05) lower than CR, and the 75 µg/mL and 100 µg/mL concentrations exhibited significantly (*p* < 0.05) higher than the CR hydrodynamic radius (Figure 1G) and % fraction volume occupancy (Figure 1H).

The 25 µg/mL GK concentration induced significantly (*p* < 0.05) lower than the CR negative charge, whilst all other GK concentrations exhibited similarly (*p* > 0.05) to the CR negative charge (Figure 1I). All GK concentrations induced similarly (*p* > 0.05) to the CR polydispersity index (Figure 1J) and were significantly (*p* < 0.05) higher than the CR hydrodynamic radius (Figure 1K) and % fraction volume occupancy (Figure 1L).

All GX concentrations induced significantly (*p* < 0.05) higher than the CR negative charge (Figure 1M), polydispersity index (Figure 1N), hydrodynamic radius (Figure 1O), and % fraction volume occupancy (Figure 1P).

### 3.2. WS1 Skin Fibroblast Morphology, Viability, Proliferation, and Metabolic Activity Assessment as a Function of the MMC Agents Used

None of the MMC agents assessed affected cell morphology (Figure S1) and viability (Figure S2). With respect to cell proliferation, via DNA quantification, only the CR at day 3, the 25 µg/mL GK at day 5, and the 50 µg/mL GX at day 5 exhibited significantly (*p* < 0.05) lower DNA than the -MMC group at the respective time point (Figure S3). Cell metabolic activity was not affected (*p* > 0.05) as a function of MMC, considering that all MMC agents had % alamarBlue^®^ reduced ranging from 95% to 109% in relation to 100% of the -MMC control group (Figure S4).

### 3.3. WS1 Skin Fibroblast Collagen Type I Deposition Assessment via Electrophoresis as a Function of the MMC Agents Used

**Figure 2:**
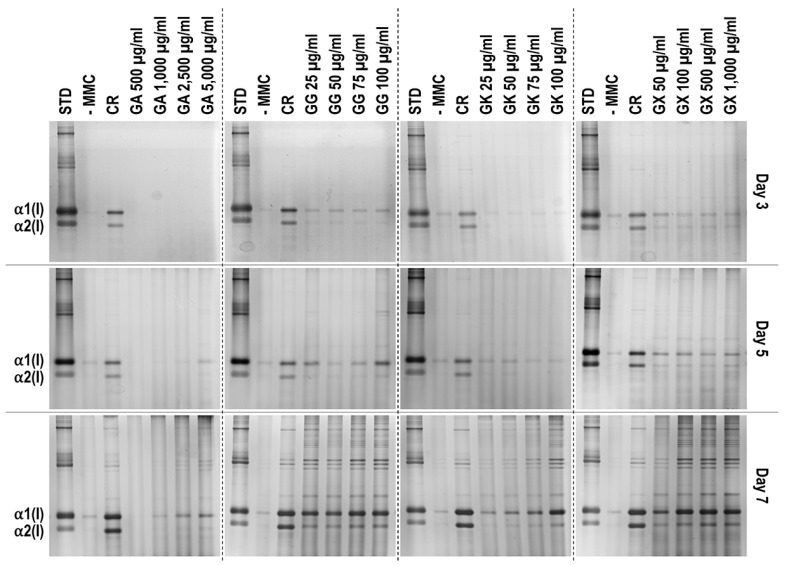
Indicative electrophoresis gels of WS1 skin fibroblast layers after 3, 5, and 7 days in culture without and with MMC (carrageenan (CR) at 75 μg/mL; gum Arabic (GA) at 500, 1000, 2500, and 5000 μg/mL; gum gellan (GG) at 25, 50, 75, and 100 μg/mL; gum karaya (GK) at 25, 50, 75, and 100 μg/mL; and gum xanthan (GX) at 50, 100, 500, and 1000 μg/mL). STD: collagen type I standard. N = 3.

**Figure 3:**
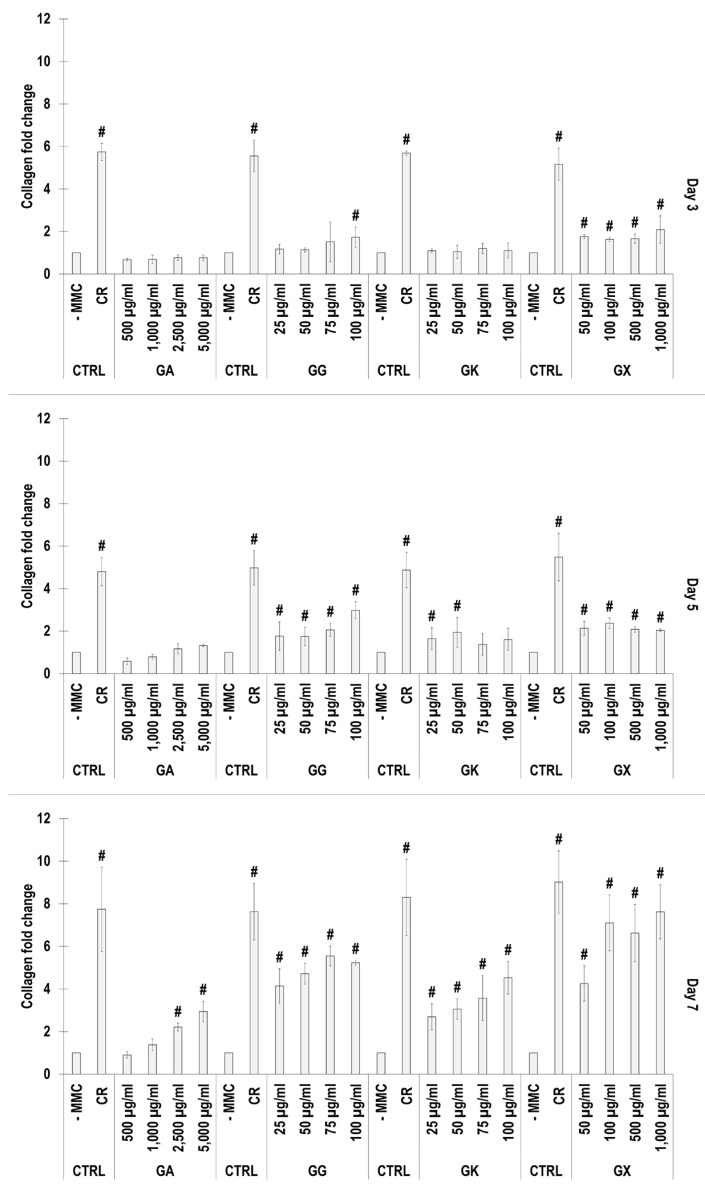
Densitometry analysis of electrophoresis gels of WS1 skin fibroblast layers after 3, 5, and 7 days in culture without and with MMC (carrageenan (CR) at 75 μg/mL; gum Arabic (GA) at 500, 1000, 2500, and 5000 μg/mL; gum gellan (GG) at 25, 50, 75, and 100 μg/mL; gum karaya (GK) at 25, 50, 75, and 100 μg/mL; and gum xanthan (GX) at 50, 100, 500, and 1000 μg/mL). # Indicates significantly (*p* < 0.05) higher population to -MMC. N = 3.

SDS-PAGE (Figure 2) and complementary densitometric analysis (Figure 3) revealed that CR at all time points induced the highest (*p* < 0.05) collagen type I deposition. Only the 2500 µg/mL and 5000 µg/mL GA concentrations at day 7 induced significantly (*p* < 0.05) higher than the -MMC group collagen type I deposition. All GG concentrations at day 5 and day 7 induced significantly (*p* < 0.05) higher than the -MMC group collagen type I deposition. All GK concentrations at day 7 induced significantly (*p* < 0.05) higher than the -MMC group collagen type I deposition. All GX concentrations at all time points induced significantly (*p* < 0.05) higher than the -MMC group collagen type I deposition.

### 3.4. WS1 Skin Fibroblast Collagen Type I, Collagen Type III, and Collagen Type V Deposition Assessment via Immunofluoresence as a Function of the MMC Agents Used

**Figure 4:**
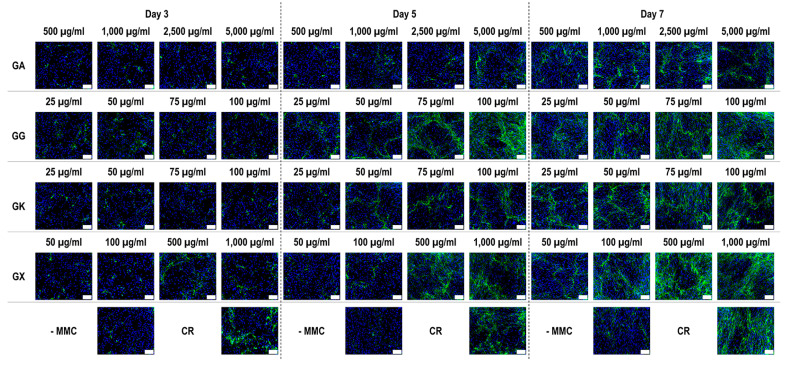
Collagen type I immunofluorescence of WS1 skin fibroblast layers after 3, 5, and 7 days in culture without and with MMC (carrageenan (CR) at 75 µg/mL; gum Arabic (GA) at 500, 1000, 2500, and 5000 µg/mL; gum gellan (GG) at 25, 50, 75, and 100 µg/mL; gum karaya (GK) at 25, 50, 75, and 100 µg/mL; and gum xanthan (GX) at 50, 100, 500, and 1000 µg/mL). Collagen type I: green. Hoechst 33342 Fluorescent stained nuclei: blue. N = 9. Scale bar: 100 µm.

**Figure 5:**
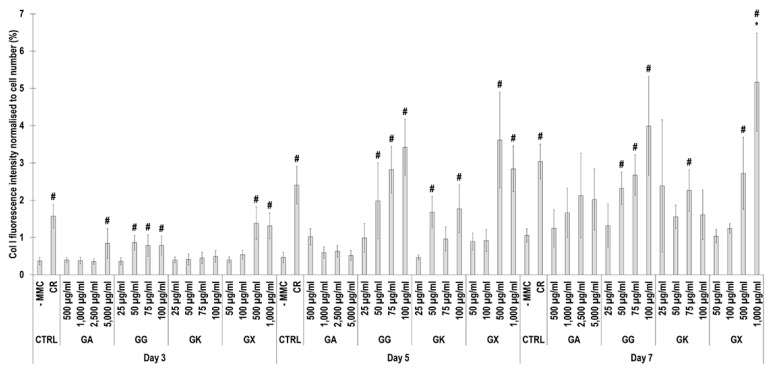
Collagen type I fluorescence intensity analysis normalised to cell number (%) of WS1 skin fibroblast layers after 3, 5, and 7 days in culture without and with MMC (carrageenan (CR) at 75 μg/mL; gum Arabic (GA) at 500, 1000, 2500, and 5000 μg/mL; gum gellan (GG) at 25, 50, 75, and 100 μg/mL; gum karaya (GK) at 25, 50, 75, and 100 μg/mL; and gum xanthan (GX) at 50, 100, 500, and 1000 μg/mL). # Indicates significantly (*p* < 0.05) higher population to -MMC. * Indicates significantly (*p* < 0.05) higher population to CR. N = 9.

Immunofluorescence (Figure 4) and complementary fluorescence intensity analysis (Figure 5) for collagen type I revealed that CR significantly (*p* < 0.05) increased collagen type I deposition at all time points, in comparison to the -MMC group at the same time point. With respect to GA, only the 5000 µg/mL concentration significantly (*p* < 0.05) increased collagen type I deposition at day 3, in comparison to the -MMC group at the same time point. The 50 µg/mL, 75 µg/mL, and 100 µg/mL concentrations of GG at all time points significantly (*p* < 0.05) increased collagen type I deposition, in comparison to the -MMC group at the respective time point. The GK concentrations of 50 µg/mL and 100 µg/mL at day 5 and the 75 µg/mL at day 7 significantly (*p* < 0.05) increased collagen type I deposition, in comparison to the -MMC group at the respective time point. With respect to GX, the 500 µg/mL and the 1000 µg/mL concentrations significantly (*p* < 0.05) increased collagen type I deposition at all time points, all in comparison to the -MMC group at a given time point. At day 3 and day 5, none of gums assessed induced significantly (*p* < 0.05) higher than the CR collagen type I deposition, whilst at day 7, only the 1000 µg/mL GX induced significantly (*p* < 0.05) higher than CR collagen type I deposition.

**Figure 6:**
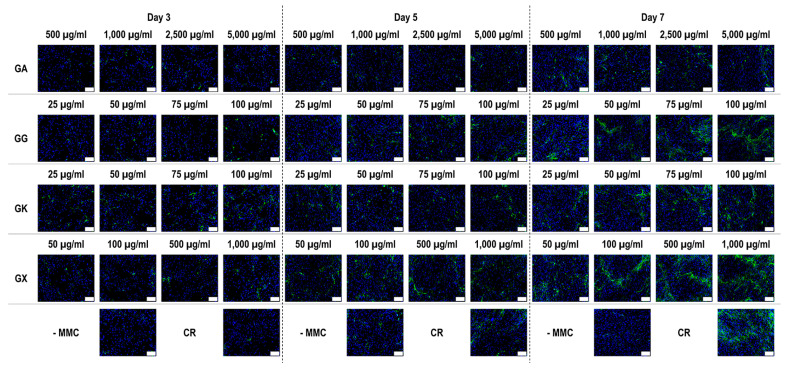
Collagen type III immunofluorescence of WS1 skin fibroblast layers after 3, 5, and 7 days in culture without and with MMC (carrageenan (CR) at 75 µg/mL; gum Arabic (GA) at 500, 1000, 2500, and 5000 µg/mL; gum gellan (GG) at 25, 50, 75, and 100 µg/mL; gum karaya (GK) at 25, 50, 75, and 100 µg/mL; and gum xanthan (GX) at 50, 100, 500, and 1000 µg/mL). Collagen type I: green. Hoechst 33342 Fluorescent stained nuclei: blue. N = 9. Scale bar: 100 µm.

**Figure 7:**
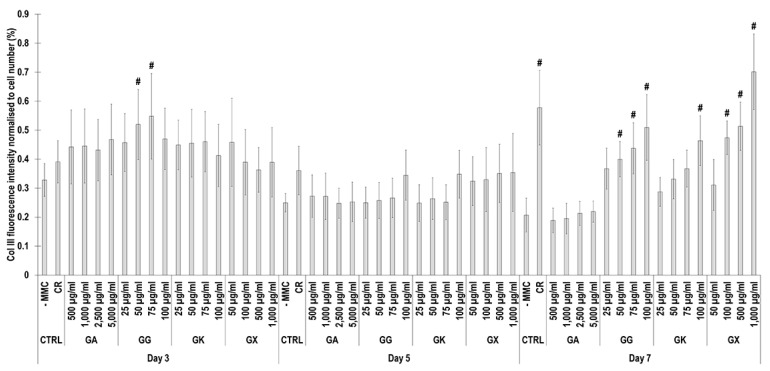
Collagen type III fluorescence intensity analysis normalised to cell number (%) of WS1 skin fibroblast layers after 3, 5, and 7 days in culture without and with MMC (carrageenan (CR) at 75 μg/mL; gum Arabic (GA) at 500, 1000, 2500, and 5000 μg/mL; gum gellan (GG) at 25, 50, 75, and 100 μg/mL; gum karaya (GK) at 25, 50, 75, and 100 μg/mL; and gum xanthan (GX) at 50, 100, 500, and 1000 μg/mL). # Indicates significantly (*p* < 0.05) higher population to -MMC. N = 9.

Immunofluorescence (Figure 6) and complementary fluorescence intensity analysis (Figure 7) for collagen type III revealed that at day 3, only the 50 µg/mL and 75 µg/mL GG concentrations significantly (*p* < 0.05) increased collagen type III deposition, in comparison to the -MMC group at the same time point. At day 5, none of the molecules assessed significantly (*p* < 0.05) increased collagen type III deposition, in comparison to the -MMC group at the same time point. At day 7, CR concentrations; the 50 µg/mL, 75 µg/mL, and 100 µg/mL GG concentrations; the 100 µg/mL GK concentration; and the 100 µg/mL, 500 µg/mL, and 1000 µg/mL GX concentrations significantly (*p* < 0.05) increased collagen type III deposition, in comparison to the -MMC group at the same time point. At day 3, day 5, and day 7, none of the gums assessed induced significantly (*p* < 0.05) higher than the CR collagen type III deposition.

**Figure 8:**
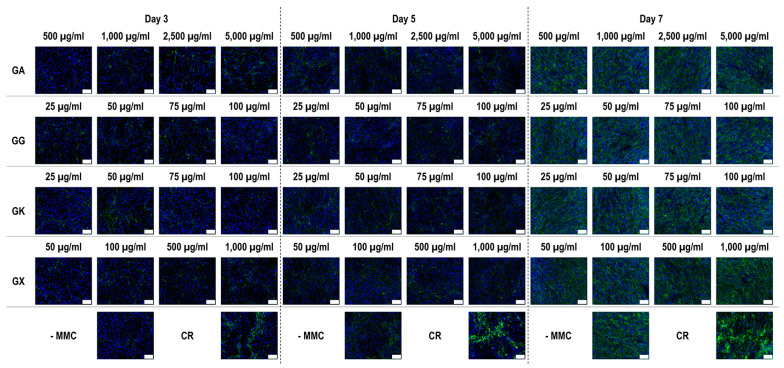
Collagen type V immunofluorescence of WS1 skin fibroblast layers after 3, 5, and 7 days in culture without and with MMC (carrageenan (CR) at 75 µg/mL; gum Arabic (GA) at 500, 1000, 2500, and 5000 µg/mL; gum gellan (GG) at 25, 50, 75, and 100 µg/mL; gum karaya (GK) at 25, 50, 75, and 100 µg/mL; and gum xanthan (GX) at 50, 100, 500, and 1000 µg/mL). Collagen type I: green. Hoechst 33342 Fluorescent stained nuclei: blue. N = 9. Scale bar: 100 µm.

**Figure 9:**
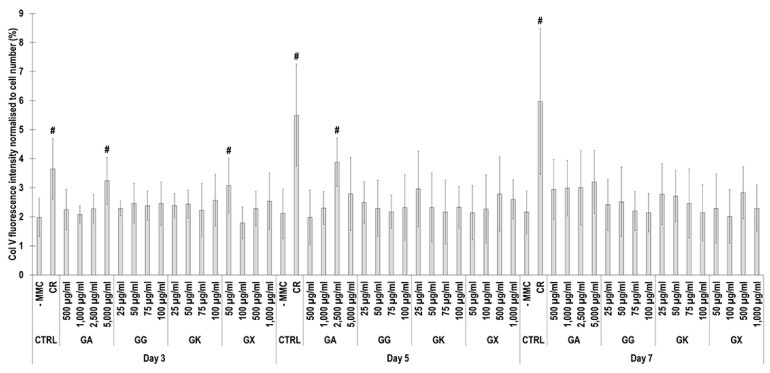
Collagen type V fluorescence intensity analysis normalised to cell number (%) of WS1 skin fibroblast layers after 3, 5, and 7 days in culture without and with MMC (carrageenan (CR) at 75 μg/mL; gum Arabic (GA) at 500, 1000, 2500, and 5000 μg/mL; gum gellan (GG) at 25, 50, 75, and 100 μg/mL; gum karaya (GK) at 25, 50, 75, and 100 μg/mL; and gum xanthan (GX) at 50, 100, 500, and 1000 μg/mL). # Indicates significantly (*p* < 0.05) higher population to -MMC. N = 9.

Immunofluorescence (Figure 8) and complementary fluorescence intensity analysis (Figure 9) for collagen type V revealed that CR significantly (*p* < 0.05) increased collagen type V deposition at all time points, in comparison to the -MMC group at the same time point. At day 3, the 5000 µg/mL GA and the 50 µg/mL GX; at day 5, the 2500 µg/mL GA; and at day 7, none of the gums assessed induced significantly (*p* < 0.05) increased collagen type V deposition, in comparison to the -MMC group at the respective time point. At day 3, day 5, and day 7, none of gums assessed induced significantly (*p* < 0.05) higher than the CR collagen type V deposition.

## 4. Discussion

Although the potential of MMC in eukaryotic cell culture has been well-established by now, the ultimate, if it exists, MMC agent is still elusive. With this in mind, we herein ventured to assess the potential of different gums (i.e., GA, GG, GK, GX) in WS1 skin fibroblast cultures and compare them to CR, the most effective, with respect to maximum ECM deposition in the shortest period of time, the MMC agent.

Starting with dynamic light scattering analysis, all the assessed macromolecules had similar properties to those that have been reported previously in the literature (e.g., CR [37], GA [64,65], GG [66,67,68,69], GK [70,71,72], GX [73,74]), considering that fluctuations among different publications may be due to system sensitivity and that dynamic light scattering data depend on concentration, temperature, ionic strength, and pH [75,76,77]. All molecules, as expected, had negative charge. CR has negative charge due to its sulphate groups [78]. GA [79,80], GG [81], and GK [82] have negative charge due to the carboxylic groups of the glucuronic acid residues. GX has high negative charge due to the presence of pyruvic acid residue and an acetyl group attached to the main chain [83]. The most notable difference was that all concentrations of GX induced significantly higher than the CR negative charge, which can be attributed to their molecular weight difference. Indeed, previous studies have shown the zeta potential to decrease as the molecular weight increases [84]. It is worth noting however that a previous study considered GX ‘medium negatively charged’ and CR ‘highly negative charged’ [85]. One should note that no values were obtained for polydispersity index and hydrodynamic radius for the 25 µg/mL GG concentration, whilst, at the same concentration, both values were measurable for GK. We attribute this to the molecular weight difference between the two molecules (i.e., 1000–2000 kDa for GG and 2000–5000 kDa for GK), considering that previous studies have shown hydrodynamic radius values to increase as molecular weight values increase [86,87,88]. At concentrations above 75 µg/mL, all macromolecules had higher than 0.68 polydispersity index values, indicative of highly dispersed populations [89]. In general, hydrodynamic radius and fractional volume occupancy, which was calculated based on hydrodynamic radius, increased with concentration, and all gums at all concentrations assessed (apart from the 25 µg/mL and 50 µg/mL GG) had higher hydrodynamic radius and fractional volume occupancy than CR and other MMC agents (e.g., dextran sulphate, polysucrose, polyvinylpyrrolidone) [33,34,37,62], but were similarly high to some seaweed polysaccharides (e.g., fucoidan, arabinogalactan, ulvan) [60]. Obviously, such high values (i.e., 201,503%) are meaningless and outside of the capacity of the system used. Indeed, dynamic light scattering experiments should be conducted at the optimal concentration for a given molecule to avoid issues with not enough light being scattered at low concentrations or multi-scattering at high concentrations when agglomeration occurs [90,91]. It is also worth noting that for fractional volume occupancy, the samples are assumed spherical, which may not be the case for these macromolecules. Considering that authors have argued that samples with polydispersity index values higher than 0.7 may be outside the capacity of dynamic light scattering [92], these measurements should be treated with caution.

With respect to cytocompatibility, as assessed by cell viability, metabolic activity, and proliferation, no notable differences appeared between -MMC and the assessed molecules; between CR and the assessed gums; and all groups supported cell growth. Although some groups resulted in reduced DNA content in comparison to the -MMC group (75 µg/mL CR at day 3, 25 µg/mL GK at day 5, and 50 µg/mL GX at day 5), we do not consider this to be significant, as it was not consistent for all time points and was not verified by cell viability and metabolic activity data. Furter, the cytocompatibility of CR has been well-established in the literature [58,93] and has even been used effectively in clinical settings [94,95]. Similarly, all assessed gums are used extensively in the biomaterials field with an equal well-documented cytocompatibility (e.g., GA [96,97,98,99], GG [100,101,102,103,104], GK [105,106,107], GX [108,109,110]) with a diverse range of cell populations.

An increased collagen type I deposition and maturation was observed for all MMC agents as a function of time in culture (as documented by increased density of α-, β-, and γ-bands on the gels), which is in accordance to previous publications [36,37]. Although none of the gums consistently outperformed CR in collagen type I and collagen type III deposition (as judged by electrophoresis and immunofluorescence analyses), we recognise that the high concentrations of GG and GX matched CR’s enhanced collagen type I and collagen type III deposition capacity at day 7. A delayed MMC effect (i.e., enhanced ECM deposition at later time point) has also been observed previously for polysucrose [31], with subsequent studies demonstrating major mesenchymal stromal effect fate consequences. Indeed, polysucrose has been shown to enhance adipogenic differentiation [26,111], whilst sulphated polysaccharides have been shown to enhance osteogenic and chondrogenic differentiation [59,60]. It will be therefore of interest to assess in future studies the mesenchymal stromal cell differentiation potential of these gums. With respect to collagen type V (a regulatory fibril-forming collagen that co-assembles with collagen type I into heterotypic fibrils [112]), it is interesting to note that only CR consistently induced increased deposition at all time points. This is of particular importance, considering that in its native state, collagen type V is degraded by metalloproteinases and gelatinases [113,114], thereby promoting physiological ECM remodelling. Moreover, previous studies have shown that the relative ratio of collagen type V to collagen type I to decrease as the ECM is matured as a function of days in culture [115], which also agrees with our data; in fact, CR induced this faster than the non-MMC group at the respective time points, further advocating the use of CR for the development of tissue engineered medicines (at day 3, day 5, and day 7, the ratios of collagen type V to collagen type I were 5.35, 4.61, and 2.05, respectively, for the -MMC group, and at day 3, day 5, and day 7, the ratios of collagen type V to collagen type I were 2.32, 2.29, and 1.96, respectively, for the CR group; all based on immunofluorescence data). We also ought to point out that previous studies have argued that polydispersity and negative charge [37] or fractional volume occupancy [62] are key modulators of enhanced and accelerated ECM deposition. With this in mind, one would have expected the GX to outperform CR in ECM deposition, which was not the case (other than the 1000 µg/mL at day 7, as judged by immunofluorescence only), but it is in agreement with previous publications, where a size-dependent MMC effect was demonstrated [116,117,118]. Evidently, a more systematic investigation on how and which physicochemical properties of the MMC agents are key modulators of ECM deposition is needed.

## 5. Conclusions

Macromolecular crowding has the potential to accelerate the development of tissue-engineered medicines. In the quest of the ideal (with respect to highest extracellular matrix deposition, in the shortest period of time without any negative effects) macromolecular crowding agent, we herein assessed the potential of gum Arabic, gum gellan, gum karaya, and gum xanthan and correlated their effect to carrageenan. Although none of the gums outperformed carrageenan, gum gellan and gum xanthan matched carrageenan’s efficiency, and therefore more detailed studies are needed to fully exploit their potential in regenerative medicine.

## Data Availability

Raw and processed data are available on reasonable request from Salomé Guillaumin, Mehmet Gurdal, and Dimitrios I. Zeugolis.

## Supplementary Materials

The following supporting information can be downloaded at: https://www.mdpi.com/article/10.3390/life14040435/s1, Figure S1: Morphology of WS1 skin fibroblasts after 3, 5, and 7 days in culture without and with the MMC agents (carrageenan (CR) at 75 µg/mL; gum Arabic (GA) at 500, 1000, 2500, and 5000 µg/mL; gum gellan (GG) at 25, 50, 75, and 100 µg/mL; gum karaya (GK) at 25, 50, 75, and 100 µg/mL; and gum xanthan (GX) at 50, 100, 500, and 1000 µg/mL) assessed. N = 9. Scale bar: 100 µm; Figure S2: Viability assessment of WS1 skin fibroblasts after 3, 5, and 7 days in culture without and with the MMC agents (carrageenan (CR) at 75 µg/mL; gum Arabic (GA) at 500, 1000, 2500, and 5000 µg/mL; gum gellan (GG) at 25, 50, 75, and 100 µg/mL; gum karaya (GK) at 25, 50, 75, and 100 µg/mL; and gum xanthan (GX) at 50, 100, 500, and 1000 µg/mL) assessed. Live cells: green. Dead cells: red. N = 6–10. Scale bar: 100 µm; Figure S3: DNA quantification of WS1 skin fibroblasts after 3, 5, and 7 days in culture without and with the MMC agents (carrageenan (CR) at 75 µg/mL; gum Arabic (GA) at 500, 1000, 2500, and 5000 µg/mL; gum gellan (GG) at 25, 50, 75, and 100 µg/mL; gum karaya (GK) at 25, 50, 75, and 100 µg/mL; and gum xanthan (GX) at 50, 100, 500, and 1000 µg/mL) assessed. * Indicates significantly (*p* < 0.05) lower population to -MMC. N = 9; Figure S4: % reduced alamarBlue^®^ quantification of WS1 skin fibroblasts after 3, 5, and 7 days in culture without and with the MMC agents (carrageenan (CR) at 75 µg/mL; gum Arabic (GA) at 500, 1000, 2500, and 5000 µg/mL; gum gellan (GG) at 25, 50, 75, and 100 µg/mL; gum karaya (GK) at 25, 50, 75, and 100 µg/mL; and gum xanthan (GX) at 50, 100, 500, and 1000 µg/mL) assessed. N = 9; Table S1: Properties of the MMC agents assessed [119,120,121,122,123,124,125,126,127]; Table S2: Solubility evaluation of the gums assessed in this study. Green background indicates soluble concentrations. Orange background indicates insoluble concentrations. N = 3.
