# Supplementary material for: Gums as Macromolecular Crowding Agents in Human Skin Fibroblast Cultures
Source: Life (Basel). 2024 Mar 25;14(4):435. doi: 10.3390/life14040435 (PMC11051389; doi:10.3390/life14040435)
Supplement: Supplementary file 1 [file life-14-00435-s001.zip › life-2878335-supplementary.pdf]

Supplementary Information

Supplementary Table S1. Properties of the MMC agents assessed.

Crowder	Properties
Carrageenan (CR)	Made up of repeating galactose units and 3,6-anhydrogalactose, both sulphated and non-sulphated. The units are joined by alternating α-1-3 and β-1-4 glycosidic linkages [119]. Molecular weight (λ carrageenan, as an example): 200-800 kDa [120].
Gum Arabic (GA)	Contains the L-arabinose, L-rhamnose, and D-glucuronic acid and 1,3-linked β-D-galactopyranosyl units. The backbone structure is mainly composed of 1,3-linked β-D-galactopyranosyl units [121]. Molecular weight: 250 kDa [122].
Gum gellan (GG)	Consists of a backbone of a repeating unit of β-D-glucose, L-rhamnose, and D-glucuronic acid and two acyl groups, acetate and glycerate, bound to the glucose residue adjacent to glucuronic acid [121]. Molecular weight: 1,000-2,000 kDa [123].
Gum karaya (GK)	A heavily acetylated polysaccharide composed of α-D-galacturonic acid and α-L-rhamnose chains. The acid groups are glycosylated with β-D-galactose or β-D-glucuronic acid residues, and about half of the rhamnose groups carry β-D-galactose units as side chains [124]. Molecular weight: 2,000-5,000 kDa [125].
Gum xanthan (GX)	Contains D-mannose, D-glucose, and D-glucuronic acid monosaccharide in molar ratios of 2:2:1. The main chain is composed of cellobiose duplicate units. The side chains include trisaccharides composed of D-mannose with the conjunction of β-(1,4)-D-glucuronic acid-β-(1,2)-D-mannose, which are connected to the main chain by α-1,3 linkages. Pyruvate acid is linked in an unknown distribution to about half of terminal D-mannose in positions 4 and 6. The D-mannose monosaccharide linked to the backbone of the polymer has an acetyl group at oxygen-6 position [126]. Molecular weight: 2,000-20,000 kDa [127].

Supplementary Table S2. Solubility evaluation of the gums assessed in this study. Green background indicates soluble concentrations. Orange background indicates insoluble concentrations. N = 3.

**Concentration (µg/ml)**	**GA**	**GG**	**GK**	**GX**
25		Assessed	Assessed	
50		Assessed	Assessed	Assessed
75		Assessed	Assessed	
100		Assessed	Assessed	Assessed
500	Assessed	X	X	Assessed
1,000	Assessed	X	X	Assessed
2,500	Assessed	X	X	X
5,000	Assessed	X	X	X
10,000	X	X	X	X
15,000	X	X	X	X
20,000	X	X	X	X
25,000	X	X	X	X

Supplementary Figure S1. Morphology of WS1 skin fibroblasts after 3, 5, and 7 days in culture without and with the MMC agents (carrageenan (CR) at 75 µg/ml; gum Arabic (GA) at 500, 1000, 2500, and 5000 µg/ml; gum gellan (GG) at 25, 50, 75, and 100 µg/ml; gum karaya (GK) at 25, 50, 75, and 100 µg/ml; and gum xanthan (GX) at 50, 100, 500, and 1000 µg/ml) assessed. N = 9. Scale bar: 100 µm.

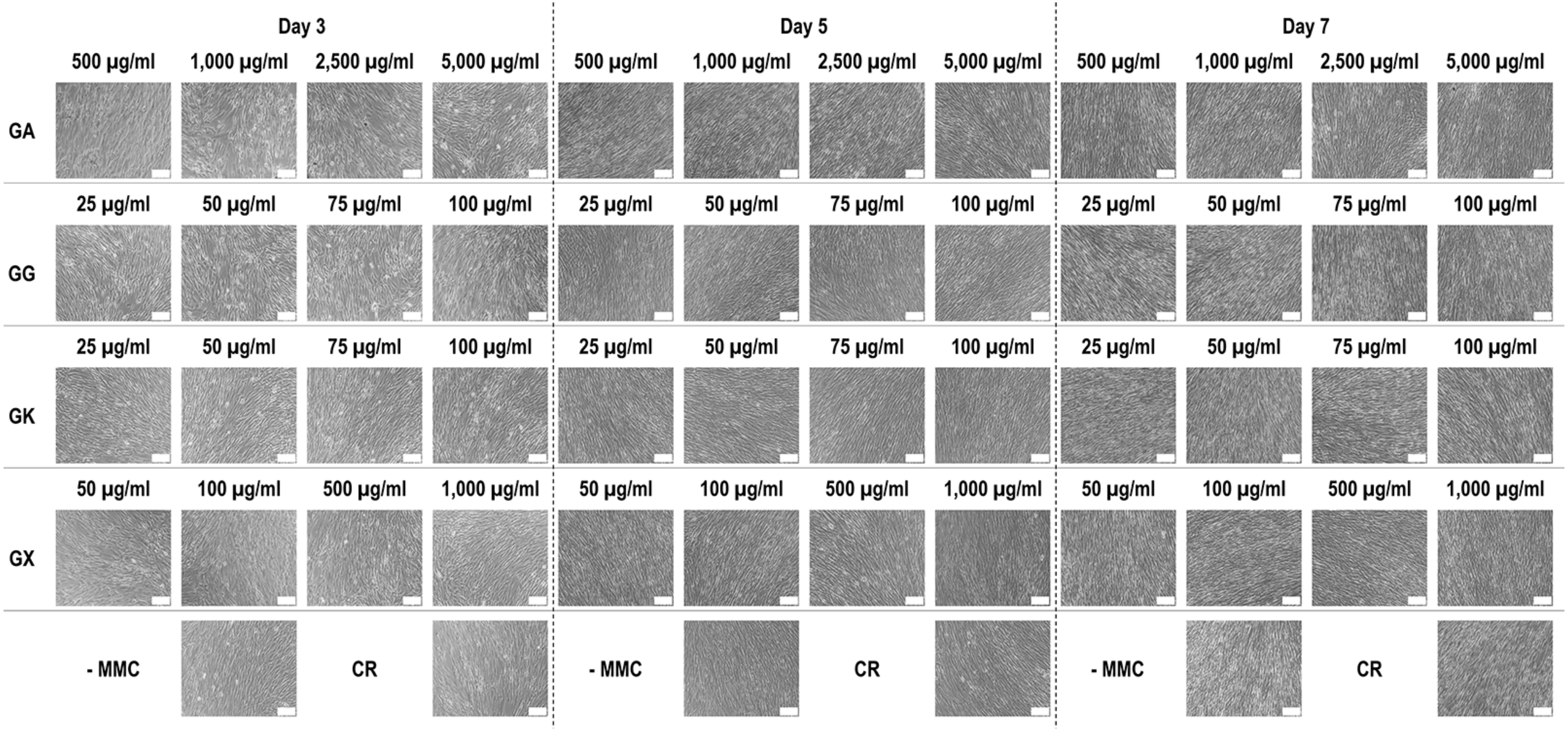

Supplementary Figure S2. Viability assessment of WS1 skin fibroblasts after 3, 5, and 7 days in culture without and with the MMC agents (carrageenan (CR) at 75 µg/ml; gum Arabic (GA) at 500, 1000, 2500, and 5000 µg/ml; gum gellan (GG) at 25, 50, 75, and 100 µg/ml; gum karaya (GK) at 25, 50, 75, and 100 µg/ml; and gum xanthan (GX) at 50, 100, 500, and 1000 µg/ml) assessed. Live cells: green. Dead cells: red. N = 6-10. Scale bar: 100 µm.

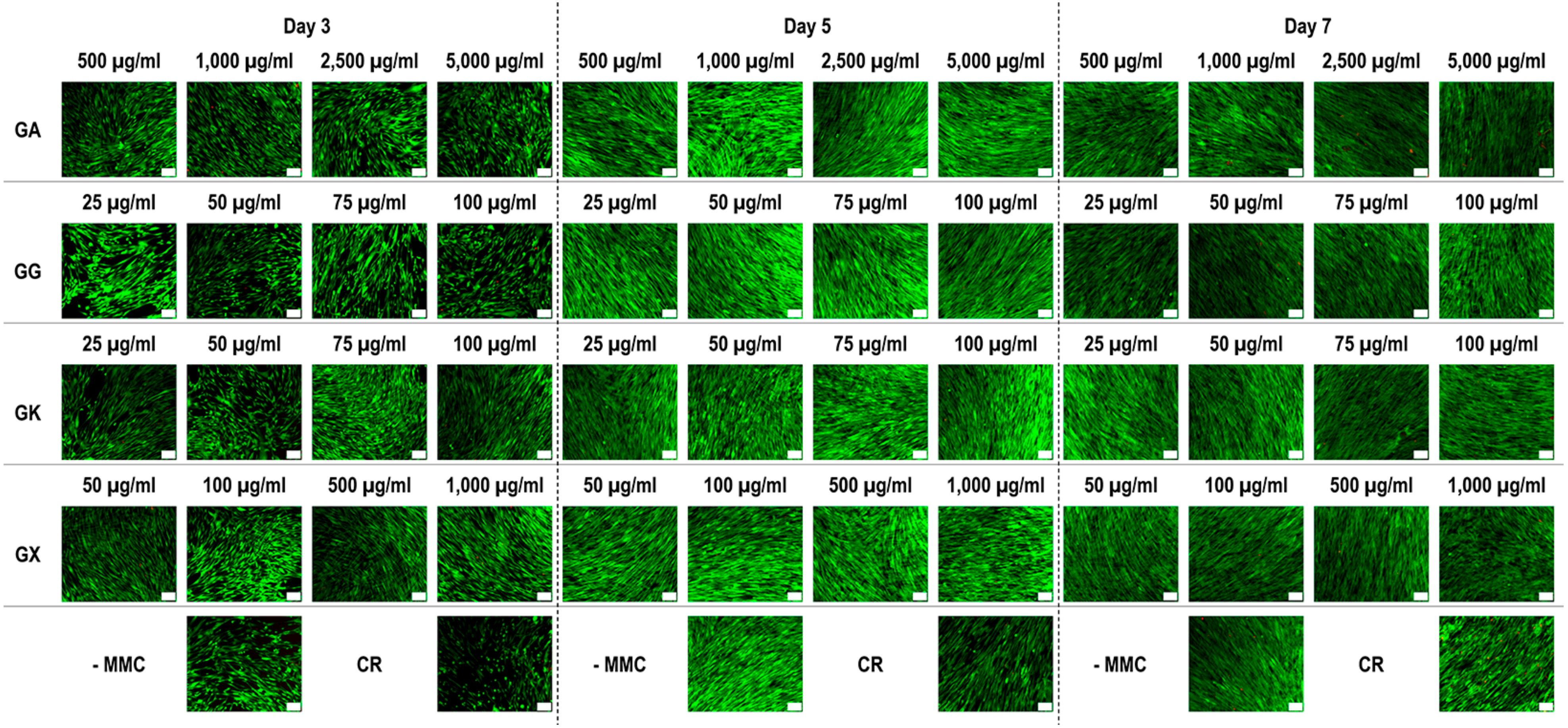

Supplementary Figure S3. DNA quantification of WS1 skin fibroblasts after 3, 5, and 7 days in culture without and with the MMC agents (carrageenan (CR) at 75 µg/ml; gum Arabic (GA) at 500, 1000, 2500, and 5000 µg/ml; gum gellan (GG) at 25, 50, 75, and 100 µg/ml; gum karaya (GK) at 25, 50, 75, and 100 µg/ml; and gum xanthan (GX) at 50, 100, 500, and 1000 µg/ml) assessed. * indicates significantly ($p < 0.05$) lower population to -MMC. N = 9.

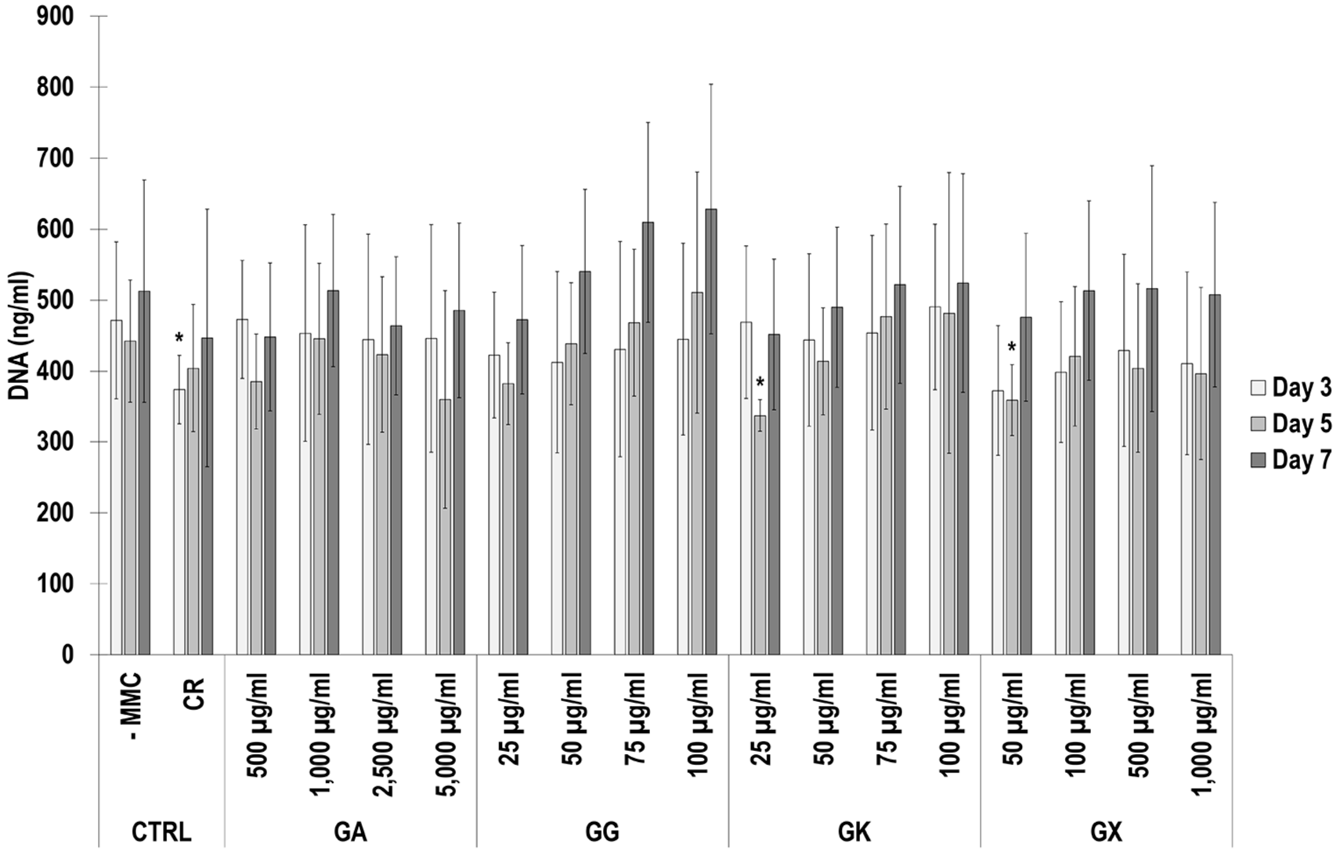

Supplementary Figure S4. % reduced alamarBlue® quantification of WS1 skin fibroblasts after 3, 5, and 7 days in culture without and with the MMC agents (carrageenan (CR) at 75 µg/ml; gum Arabic (GA) at 500, 1000, 2500, and 5000 µg/ml; gum gellan (GG) at 25, 50, 75, and 100 µg/ml; gum karaya (GK) at 25, 50, 75, and 100 µg/ml; and gum xanthan (GX) at 50, 100, 500, and 1,000 µg/ml) assessed. N = 9.

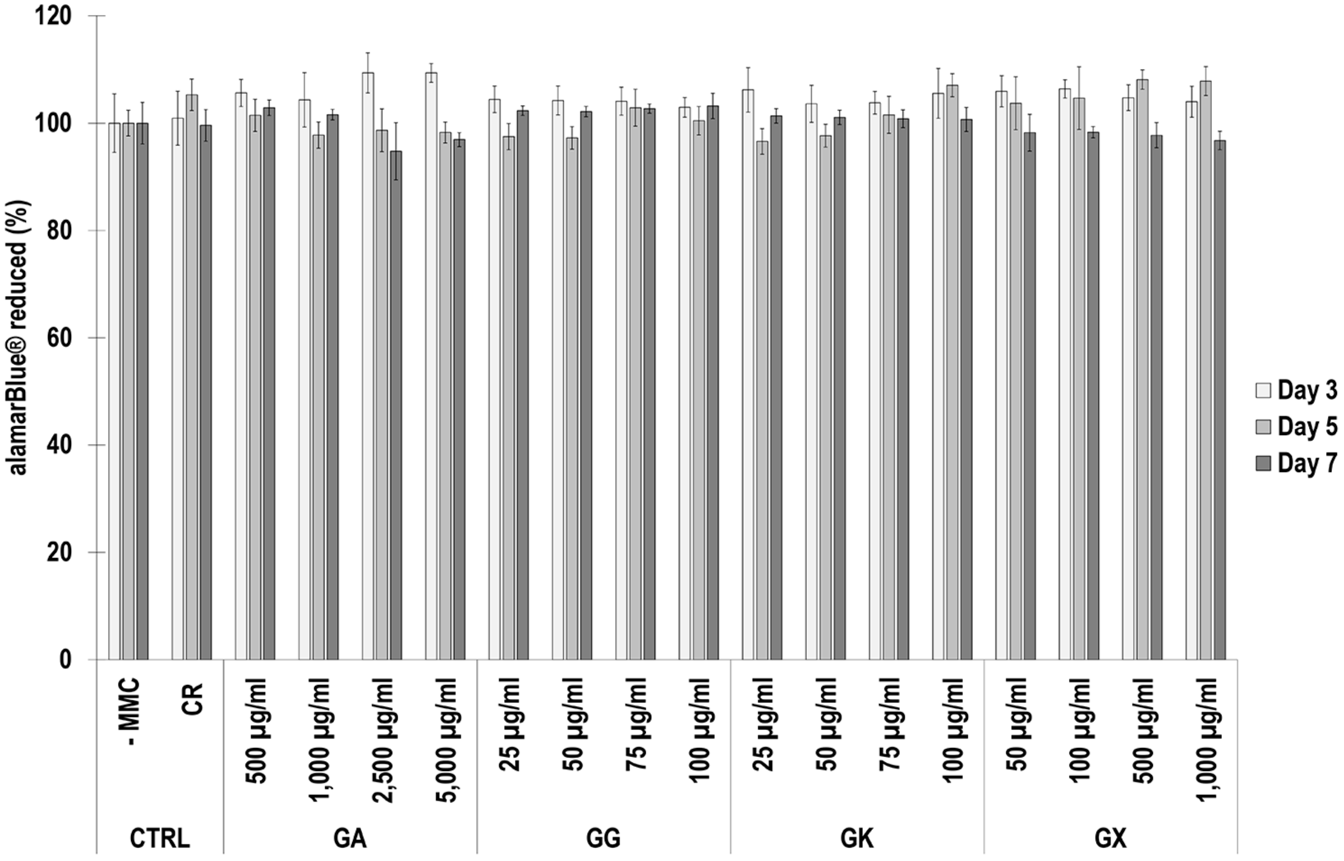

References

119. Kulkarni, V.S.; Shaw, C. (Eds.) Chapter 5—Use of Polymers and Thickeners in Semisolid and Liquid Formulations. In *Essential Chemistry for Formulators of Semisolid and Liquid Dosages*; Academic Press: Boston, MA, USA, 2016; pp. 43–69.
120. Buecker, S.; Grossmann, L.; Loeffler, M.; Leeb, E.; Weiss, J. High molecular weight λ-carrageenan improves the color stability of phycocyanin by associative interactions. *Front. Sustain. Food Syst.* **2022**, *6*, 915194. https://doi.org/10.3389/fsufs.2022.915194.
121. Dave, P.N.; Gor, A. Chapter 3—Natural Polysaccharide-Based Hydrogels and Nanomaterials: Recent Trends and Their Applications. In *Handbook of Nanomaterials for Industrial Applications*; Hussain, C.M., Ed.; Elsevier: Amsterdam, The Netherlands, 2018; pp. 36–66.
122. Swenson, H.; Kaustinen, H.; Kaustinen, O.; Thompson, N. Structure of gum arabic and its configuration in solution. *J. Polym. Sci. A-2 Polym. Phys.* **1968**, *6*, 1593–1606.
123. Sudhakar, Y.N.; Selvakumar, M.; Bhat, D.K. (Eds.) Chapter 4—Biopolymer Electrolytes for Solar Cells and Electrochemical Cells. In *Biopolymer Electrolytes*; Elsevier: Amsterdam, The Netherlands, 2018; pp. 117–149.
124. Williams, P.A.; Phillips, G.O. GUMS|Properties of Individual Gums. In *Encyclopedia of Food Sciences and Nutrition*, 2nd ed.; Caballero, B., Ed.; Academic Press: Oxford, UK, 2003; pp. 2992–3001.
125. Padil, V.; Wacławek, S.; Černík, M.; Varma, R. Tree gum-based renewable materials: Sustainable applications in nanotechnology, biomedical and environmental fields. *Biotechnol. Adv.* **2018**, *36*, 1984–2016.
126. Nasrollahzadeh, M.; Sajjadi, M.; Nezafat, Z.; Shafiei, N. Chapter 3–Polysaccharide biopolymer chemistry. In *Biopolymer-Based Metal Nanoparticle Chemistry for Sustainable Applications*; Nasrollahzadeh, M., Ed.; Elsevier; Amsterdam, The Netherlands, 2021; pp. 45–105.
127. Hu, X.; Wang, K.; Yu, M.; He, P.; Qiao, H.; Zhang, H.; Wang, Z. Characterization and antioxidant activity of a low-molecular-weight xanthan gum. *Biomolecules* **2019**, *9*, 730.
